# Cardiac Telocytes 16 Years on—What Have We Learned So Far, and How Close Are We to Routine Application of the Knowledge in Cardiovascular Regenerative Medicine?

**DOI:** 10.3390/ijms222010942

**Published:** 2021-10-10

**Authors:** Martin Klein, Mária Csöbönyeiová, Stanislav Žiaran, Ľuboš Danišovič, Ivan Varga

**Affiliations:** 1 National Institute of Rheumatic Diseases, Nábrežie I. Krasku 4, 921 12 Piešťany, Slovakia; stanoziaran@gmail.com (S.Ž.); lubos.danisovic@fmed.uniba.sk (Ľ.D.); ivan.varga@fmed.uniba.sk (I.V.); 2 Institute of Histology and Embryology, Faculty of Medicine, Comenius University, Sasinkova 4, 811 08 Bratislava, Slovakia; maria.csobonyeiova@fmed.uniba.sk; 3 Department of Urology, Faculty of Medicine, Comenius University, Limbová 5, 833 05 Bratislava, Slovakia; 4 Institute of Medical Biology, Genetics and Clinical Genetics, Faculty of Medicine, Comenius University, Sasinkova 4, 811 08 Bratislava, Slovakia

**Keywords:** cardiac telocytes, regeneration, repair, myocardial infarction, transplantation, stem cells

## Abstract

The regeneration of a diseased heart is one of the principal challenges of modern cardiovascular medicine. There has been ongoing research on stem-cell-based therapeutic approaches. A cell population called telocytes (TCs) described only 16 years ago largely contributed to the research area of cardiovascular regeneration. TCs are cells with small bodies and extremely long cytoplasmic projections called telopodes, described in all layers of the heart wall. Their functions include cell-to-cell signaling, stem-cell nursing, mechanical support, and immunoregulation, to name but a few. The functional derangement or quantitative loss of TCs has been implicated in the pathogenesis of myocardial infarction, heart failure, arrhythmias, and many other conditions. The exact pathomechanisms are still unknown, but the loss of regulative, integrative, and nursing functions of TCs may provide important clues. Therefore, a viable avenue in the future modern management of these conditions is TC-based cell therapy. TCs have been previously transplanted into a mouse model of myocardial infarction with promising results. Tandem transplantation with stem cells may provide additional benefit; however, many underresearched areas need to be addressed in future research before routine application of TC-based cell therapy in human subjects. These include the standardization of protocols for isolation, cultivation, and transplantation, quantitative optimization of TC transplants, cost-effectivity analysis, and many others.

## 1. Introduction

Ischemic heart disease is at the top tier of unflattering global health statistics. Despite an unparalleled achievement in diagnostic and therapeutic measures over the recent years, which have caused a decline in mortality, it is the leading cause of death around the globe, nevertheless. In Europe, 20% of all deaths are attributed to ischemic heart disease [1]. The researchers had long thought that the limited intrinsic regenerative capacity of the human myocardium is a hurdle, which is hard to overcome; thus, heart transplantation in patients with heart failure would be the most viable prospect for a long time. Fortunately, the advancements in molecular biology, tissue engineering, and cell-based therapy have made cardiac repair and regeneration more tangible and attainable than ever [2].

There are two main strategies for achieving these goals: the first is the implementation of different types of stem cells, most notably embryonic stem cells and induced pluripotent stem cells, with promising results from animal studies. The second approach is the targeting of endogenous pathways in cardiomyocytes themselves, via which they can acquire the regenerative capacity seen in lower animals, e.g., zebrafish [3]. A novel field of study is the use of immune cell-derived exosomes, which could aid in regeneration by suppressing excessive inflammation in the damaged myocardium [4]. Needless to say, all these optimistic outlooks still face many unresolved issues, which have to be worked out before routine clinical application.

One cell population has been largely overlooked but might be “the last piece of the puzzle” necessary for successfully integrating all the approaches mentioned above—telocytes (TCs).

The most common definition of TCs found in most papers on this peculiar cell population is that TCs are recently discovered [5] and best characterized as cells with extremely long cytoplasmic projections called telopodes [6]. Here, it is necessary to clarify some essential terminological specifics which reflect the “serendipity” of TC discovery. In 2005, Popescu et al. described cells in the pancreas and named them interstitial Cajal-like cells (ICLCs) due to superficial similarities with interstitial cells of Cajal (ICCs) [7,8]. Five years later, in 2010, Popescu and Faussone-Pellegrini proposed a new name—TCs—because their morphological and functional distinctiveness had become evident [6]. Because the firm identity of TCs was established in 2010, some may argue that TCs should be associated with this year. In any case, we think that they should no longer be described as “recently discovered” by any measure.

On the other hand, the definition of TCs as cells with telopodes [6] is entirely appropriate because the morphological features of telopodes are the most prominent and significant characteristics which distinguish TCs from other populations of interstitial cells. It is not only the length of telopodes that is distinctive. Telopodes are also specific due to their moniliform alternation of thin and thick segments termed podomers and podoms, respectively [6].

To this date, more than 450 papers on TCs have been indexed in Web of Science, SCOPUS, or Medline/PubMed databases. These papers have covered almost all the aspects of TCs, including their physiological functions, molecular characteristics, and role in the pathogenesis of countless diseases in virtually all organs of the human body and many different animal species [9,10,11]. They have been described in the pancreas [12], liver [13], uterus [14], ovaries [15], uterine tubes [16], skin [17], kidney [18], eye [19], lungs [20], gallbladder [21], gut [22,23,24], and urinary bladder [25], as well as in surprising locations such as the teat of a cow [26], and lastly, yet importantly in the heart [27,28,29], with cardiac TCs being one of the most studied and known. It is not at all surprising, considering the abovementioned epidemiology of ischemic heart disease.

This review article aims to summarize all the knowledge on cardiac TCs concisely from their initial description to this date, with the main focus on applications in cardiovascular regenerative medicine.

## 2. Morphology, Location, Functions, and Topographic Relations of Cardiac Telocytes

**Figure 1:**
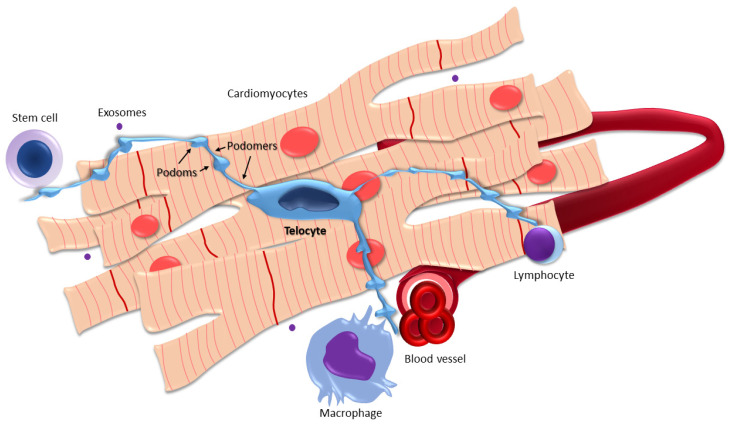
The typical morphology of a telocyte (TC) with long cytoplasmic projections—telopodes—showing alternating podomers and podoms making contacts with cardiomyocytes and components of the interstitium (e.g., immune cells, blood vessels). TCs also shed extracellular vesicles (e.g., exosomes).

The most prominent morphological features of TCs are a small inconspicuous spindle-shaped, star-shaped, triangular or oval cell body and very long, yet contrastingly thin telopodes, which make TCs difficult to study using common techniques of light microscopy, since the thickness of telopodes is roughly about the resolving power of the light microscope [30]. Due to these odd characteristics, TCs are routinely studied by immunohistochemistry, immunofluorescence, electron microscopy, or focused ion beam scanning electron microscopy, enabling 3D reconstructions [31]. From these methods, transmission electron microscopy (TEM) is the gold standard for studying TC morphology, which reveals their most typical ultrastructural characteristics. In addition to the already mentioned features (small cell body, thin telopodes), the nucleus is mainly heterochromatic, surrounded by a small amount of cytoplasm with few cell organelles. The number of telopodes ranges from 1–5 with dichotomous branching. Their length can also be considerably variable, from 10 to 1000 μm, while their thickness varies between 0.1 and 0.5 μm. It has already been outlined that telopodes have another important morphological feature apart from their extraordinary length. They look like strings of beads, resulting in the Latin-derived term moniliform. Thin podomers alternate with dilated podoms, typically containing various cell organelles, namely, mitochondria and endoplasmic reticulum. Caveolae may also be present [30]. Lastly, yet importantly, telopodes typically make homocellular and heterocellular contacts with different components of both the stroma and the parenchyma [32]. The typical morphology of a TC is depicted in Figure 1.

Although TCs do not have a specific immunophenotype, i.e., no single immunohistochemical marker can unambiguously distinguish TCs from other cell populations, immunohistochemistry is a practical and frequently employed method of TC study. One of the most cited markers of TCs is CD34. For this reason, TCs are sometimes referred to as CD34^+^ stromal cells [33]. TCs also express vimentin (implying their mesenchymal origin), α-SMA, PDGFRα, and CD117/c-kit, to name but a few. As far as cardiac TCs are concerned, CD34, PDGFRα, and CD117/c-kit are the most typical. When used in double immunostaining, Zhou et al. previously suggested that the combination of CD34 and PDGFRα might be specific for cardiac TCs [34]. TCs coexpressing these two markers were also described in a recent study authored by Lis et al. [35].

As of today, TCs have been described in all layers of the heart wall. In the endocardium, TCs were identified in the subendocardium in both the atria and the ventricles. Using the TEM, subendocardial TCs were observed to make gap junction-like contacts with fibroblasts. Moreover, the processes of TCs were in close vicinity to about one-third of endothelial cells, suggesting a role in the formation of a counterpart to the blood–brain barrier dubbed the blood–heart or blood–myocardial barrier [36]. The layer of the heart wall in which TCs are best described is the myocardium. Myocardial TCs use their long telopodes to make contacts with different components of the myocardial interstitium, but most importantly, they make heterocellular junctions with cardiomyocytes. In the epicardium, TCs were identified in the stem-cell niches, where they “nurse” cardiac progenitor cells. Gherghiceanu and Popescu performed an electron microscopic study of TCs and other cell populations inside the stem-cell niches. They found out that the loose connective tissue of the niches is highly vascularized and houses a heterogeneous population of cells, including different immune cell types and TCs. TCs were observed to support cardiomyocyte progenitor cells mechanically [37]. By establishing cell-to-cell contacts with them, TCs also mediate essential processes necessary for successfully integrating newly differentiated cardiomyocytes into the cellular microenvironment of the myocardium [38]. In addition to the direct communication via heterocellular junctions with other cells, epicardial TCs were also demonstrated to shed extracellular vesicles containing bioactive molecules, through which TCs may influence their surroundings in a paracrine manner [39]. It is important to note that TCs were also identified in stem-cell niches of other organs, including the gut, skeletal muscle, lung, and skin [40]. A significantly underresearched component of the epicardium is the epicardial white adipose tissue. This special type of visceral fat directly surrounds the epicardial branches of the coronary arteries. Multiple lines of evidence suggest that the hormonally active adipose tissue plays a role in local tissue homeostasis, whose dysregulation may negatively influence the wall of coronary arteries, leading to ischemic heart disease [41,42,43]. The knowledge on white adipose tissue TCs is scarce. In normal white adipose tissue, TCs were observed in connective tissue septa, as well as inside the lobules in close topographic relation to adipocytes, blood, and lymphatic vessels [44]. Díaz-Flores et al. also reviewed the role of adipose tissue TCs in different pathological conditions, including various adipose tissue tumors. They summarized that adipose tissue TCs might be involved in the microenvironmental regulation via intercellular signaling [44]. Unfortunately, there are no studies on TCs inside the epicardial adipose tissue, which could elucidate their roles in normal and pathological processes occurring in this clinically significant type of white adipose tissue.

## 3. Telocytes in Heart Diseases

It will be no exaggeration to say that TCs are unmatched in the vastness of their possible roles in the pathogenesis of different diseases, regardless of which animal species or organ we are referring to. In the heart alone, TCs have been studied in myocardial infarction [45], heart failure [46], arrhythmias [47], or atrial amyloidosis [48]. Their numerical alteration was also described in the heart of elderly patients, suggesting one of the many reasons why an aging heart is more disease-prone [27].

### 3.1. Myocardial Infarction

Multiple studies have demonstrated that the normal function and morphology of cardiac TCs are severely disrupted during myocardial infarction [45,49,50]. At first glance, this finding is not very surprising, given that the myocardial ischemia, with subsequent apoptosis, necrosis, and overall derangement of the cellular microenvironment, alters all the cell populations in the affected site, including TCs. It would suggest that the loss of TCs is merely a consequence of pathological processes occurring in the infarcted myocardium. It is indeed the case, but it has to be stated that previous studies found out that TCs are particularly fragile during hypoxia; hence, they are perhaps among the first cell populations negatively affected by the lack of nutrients and oxygen, leading to further derangement of normal TC-dependent myocardial architecture and function [45]. The most dreaded consequence of myocardial infarction in those patients who survive is the loss of contractile tissue, which is replaced by functionally inferior connective tissue scar, resulting in the loss of inotropic capacity of the heart, eventually progressing to heart failure [51]. The current research shows that TCs may also play a role in the development of these deleterious outcomes, not only in the pathogenesis of the myocardial infarction itself. This is documented by the experimental data, which indicate that TCs can be negatively influenced by dynamical changes in the composition of the extracellular matrix during the reparation process [52]. Moreover, according to a study of TCs and their involvement in the pathogenesis of fibrotic remodeling of the colonic wall in ulcerative colitis, it has been hypothesized that the loss of TCs may lead to deregulation of fibroblast to myofibroblast transition [53]. This transition also promotes cardiac fibrosis since myofibroblasts are excessively active in the synthesis of the extracellular matrix components [54]. The loss of TCs was also described in myocardial fibrotic lesions in patients diagnosed with systemic sclerosis, underscoring the importance of TCs in normal tissue maintenance [55].

### 3.2. Arrhythmia

Arrhythmias of different etiologies have also been discussed in relation to the possible role of TCs in their development. It is not at all surprising, given that TC discovery is closely linked to ICCs—pacemaker cells of the gut. TCs, formerly known as ICLCs, have been previously described in various organs participating in electrophysiological processes, including mechanoelectrical transduction and pacemaking [56]. DeSimone et al. found out that myocardial TCs of a dog express anoctamin-1. This voltage-gated calcium-activated anion channel is also found in ICCs as a major ion channel responsible for their pacemaker function. These results suggest that electrophysiological regulation and, thus, potential significance in arrhythmogenesis could be linked to myocardial TCs [57]. Not only that, Mitrofanova et al. also described TCs in the human sinoatrial node in close vicinity to pacemaker cells and contractile cardiac muscle cells using immunohistochemistry, confocal laser scanning microscopy, and TEM. The authors hypothesized about the modulating effects of TCs, although conclusive knowledge on their precise role has not been obtained. Future research may elucidate the role of TCs in the regulation of cardiac electrophysiology in the sinoatrial node—the top tier of normal heart rhythm [58]. Back in 2008, when TCs were known only for about 3 years and still referred to as ICLCs, Gherghiceanu et al. demonstrated the presence of TCs in the myocardial sleeves of the human pulmonary veins. According to their immunohistochemical and ultrastructural analysis of TCs in this location, the authors assumed that TCs may act as yet unrecognized agents in the pathogenesis of atrial fibrillation. This assumption was made according to the observation of the TC-derived 3D interstitial network among different components of the myocardial sleeve, including blood vessels, nerves, and cardiomyocytes. Since it is known that the myocardial sleeves can be a source of ectopic beats which can initiate the atrial fibrillation, the authors pondered on the possibility of implicating TCs in this pathological condition [59].

## 4. Cardiac Telocytes in Cardiovascular Regenerative Medicine—Recent Developments

The potential of cardiac TCs as important players in cardiovascular regeneration is substantiated by the research focused on the integrative, regulative, and nursing functions of TCs in the heart wall. As mentioned earlier, one of the most promising findings is that TCs are located in the epicardial stem-cell niches. Bei et al. comprehensively reviewed the role of TCs in the homeostatic regulation of the whole stem-cell niche, where they are capable of regulating the functional characteristics, dynamics, commitment, and other aspects of stem-cell physiology. In addition to stem cells, the authors provided an excellent overview of the other important heterocellular connections imperative for successful TC-mediated cardiac regeneration. These include connections with cardiomyocytes and different populations of interstitial cells, including fibroblasts, immune cells, pericytes, and endothelial cells, which are also important in the whole orchestration of the regeneration and repair of the heart [60].

For a better understanding of the role of TCs during repair and regeneration, the data from embryonic studies can provide valuable insights. Faussone-Pellegrini and Bani conducted an immunohistochemical and electron microscopic study of a mouse myocardium during the E14 and E17 stages of embryonic development. According to the morphological findings of TCs in close vicinity to immature components of the developing heart, the authors concluded that TCs possibly perform significant tasks during cardiogenesis, including the organization of the cytoarchitecture of the myocardial constituents, mechanical support, and supervision of the correct sequence of stem-cell differentiation [61]. Papers focused on congenital heart defects could also provide additional knowledge. A recent study performed on samples from patients with tetralogy of Fallot revealed that TCs might coordinate the differentiation of stem cells and use paracrine signaling to regulate all the surrounding interstitial compartment [62].

Experimental data also shed light on the prospects of the actual use of TCs in human regenerative medicine. A recent original article by Liao et al. presented a yet unknown mechanism of the regenerative potential of cardiac TCs. The authors found out that cardiac TCs produce exosomes containing miRNA-21-5p, which can inhibit apoptosis in cardiac microvascular endothelial cells. It is essential in the process of angiogenesis, which is necessary for favorable regeneration after myocardial infarction. These paracrine-acting molecules show another fascinating future avenue in the form of cell-free therapy, which would not require the cells themselves since it has been progressively clear that, in many instances, the regeneration happens not via a direct proliferation and differentiation of stem cells but via paracrine signaling through molecules from cell-derived structures (e.g., exosomes) [63]. Cardiac TCs also use other types of extracellular vesicles, namely, ectosomes and multivesicular cargos. Multiple lines of evidence show that these vesicles may epigenetically modulate the physiology of cardiac stem cells, which themselves produce signaling molecule-containing extracellular vesicles, thus reciprocally influencing the cardiac TCs. This not yet entirely understood crosstalk between stem cells and TCs may be the key to fully embracing the regenerative potential of TCs [64,65].

In addition to the paracrine action mediated by extracellular vesicles, TCs make direct cell-to-cell contacts with cardiac stem cells, as observed by Popescu et al., who established a coculture of these two cell populations. They formed classic cell-to-cell junctions but also unusual junctions such as puncta adherentia and stromal synapses. The authors recognized a significant obstacle in the successful implementation of cell therapy in cardiovascular regenerative medicine. The tissue, e.g., after myocardial infarction, has specific characteristics resulting from the damage associated with the pathological process, making it especially challenging for the grafted cells to survive in this inhospitable microenvironment. These include ischemia, inflammation, proapoptotic signaling, and disintegrated extracellular matrix. Therefore, it is vital to thoroughly understand the exact interaction of stem cells and TCs through in vitro reproduction of the processes occurring in the stem-cell niches. Perhaps this is an inevitable step for successfully applying the cell therapy in vivo [66].

In elucidating the role of TCs in cardiac regeneration, a highly valuable course of research is that focused on animals with almost perfect cardiac regenerative capacity. One such experimental paper was published in 2020, describing TCs in the heart of the western clawed frog (*Xenopus tropicalis*). Lv et al. used electron microscopy and immunofluorescence to visualize TCs in this species. Moreover, they surgically removed the apex of the frog’s heart to study cardiac TCs on this injured site. Interestingly, after 8 days, the damaged TCs were among the first cells to regenerate fully. This quick recovery suggests that TC renewal might be the first essential step in any further fruitful regeneration of the tissue architecture ad integrum [67]. As mentioned earlier, the loss of TCs is an important pathogenetic moment in the development of myocardial infarction. In order to study the possible ways to mitigate its progression and/or to alleviate its potential adverse outcomes, Zhao et al. transplanted TCs into the infarction zone, as well as the border zone. They found out that such a procedure provided a significant benefit in terms of the reduction in the infarction size and improved functional aspects of the myocardium [45]. TC transplantation has also been performed in other organs. Zheng et al. found out that transplanted TCs could mitigate the induced renal fibrosis in rats [68]. Focusing on TCs in the respiratory system, Zhang et al. established a rat model of acute lung injury and observed the effect of cotransplantation of TCs with mesenchymal stem cells. The results demonstrated that TCs had a synergic effect on the experimental lung injury’s attenuation [69]. TCs were also previously implicated in the pathogenesis of a broad spectrum of chronic inflammatory and fibrotic diseases, including Crohn’s disease, liver fibrosis, and psoriasis. TC transplantation, either in a solitary fashion or together with stem cells, was also discussed as a promising future avenue in the state-of-the-art therapeutic management of these conditions [70].

The importance of TCs in cardiac regeneration was also recognized in studies whose research goal was to scrutinize other cell populations with regenerative capabilities. Miao et al. attempted to investigate the effect of the transplantation of induced pluripotent stem cell-derived mesenchymal stem cells in a mouse model of myocardial infarction. Although TCs were not the prime experimental focus in this study, the authors found out that the beneficial effect of the transplanted cells was reinforced by TCs, which contributed to supporting the tissue architecture, mechanotransduction, and elasticity [71]. Similar research was conducted by Ja et al., whose experimental study also involved the induction of pluripotent stem cells, with further differentiation to cardiomyocyte progenitors and cardiomyocytes (induced pluripotent stem cells were differentiated into mesenchymal stem cells in the previous study). They also transplanted the cells into the infarcted myocardium of an animal model. In the group of animals that received the cardiac progenitor cell transplant, the authors observed an improvement in myocardial function. It was correlated with increased angiogenesis and an enhanced network of cardiac TCs in the zone of myocardial infarction [72]. These results suggest that, in the case of TC absence or dysfunction, the cells responsible for cardiac regeneration cannot perform their tasks efficiently. Therefore, TCs should always be considered when researching the cardiac reparative processes.

## 5. Controversies in Telocyte Research

It is necessary to point out one peculiar fact about TCs. They are still considered controversial among scientists. Many do not recognize them as an individual cell population. To the best of our knowledge, TCs are still not included in any histological textbook, histology curricula at the universities, or the official histological nomenclature *Terminologia Histologica* [73]. In our previous paper, we proposed to include TCs into the second edition of this official nomenclature and histology textbooks, so that TCs will become more widely known among scholars and students [74]. Some research teams have actively challenged previous experimental data on TCs in different organs. For instance, Rusu et al. were critical about the true nature of putative TCs in different organs. In the heart, they discussed the possibility that what were previously described as TCs were actually endothelial cells or lymphatic blood vessels caught in longitudinal section, thus resembling telopodes. They substantiated their suspicion by the absence of immunohistochemical differentiation of TCs from lymphatic system components by using specific markers of lymphatic endothelial cells, e.g., LYVE-1 or podoplanin [75]. These objections were recently addressed and experimentally debunked. Rosa et al. performed an immunofluorescent study that clearly demonstrated that TCs are different from lymphatic endothelial cells. TCs were CD34^+^/PDGFRα^+^/podoplanin^−^/LYVE-1^−^, while lymphatic endothelial cells were CD34^−^/PDGFRα^−^/podoplanin^+^/LYVE-1^+^. This comparative study not only demonstrated the uniqueness of TCs, it also showed that TCs possibly regulate microlymphatic circulation [76]. Another successfully implemented approach to distinguish TCs from other interstitial components is the immunostaining for CD31. When a tissue section is double-stained for CD31 and CD34, it is easy to differentiate endothelial cells lining the blood vessels, which are CD34^+^/CD31^+^, from CD34^+^/CD31^−^ TCs [77]. Hostiuc et al. also authored a discerning systematic review of papers on TCs, which analyzed and summarized six experimental studies focused on cardiac TCs during myocardial infarction. The authors were unable to find definitive evidence of a causal link between TCs and different aspects of the pathogenesis of myocardial infarction. Their objections were mainly rooted in the inability of the studies in question to unambiguously distinguish TCs from other cell populations with akin morphological characteristics and immunophenotypes [78]. To avoid such ambiguity, the abovementioned double-staining approach should always be considered. We also authored a paper focused on different aspects of TC research regarding their uniqueness as an individual cell population. In studies using in vitro cultivation as the method of TC study, they can sometimes be mistaken for long-term cultured fibroblasts or stem cells which form cytoplasmic projections similar to telopodes [79]. On the other hand, there is a substantive body of experimental papers which specifically focused on the differentiation of TCs from other interstitial components. Bei et al. established a cell culture of TCs and fibroblasts where they described immunophenotypic differences between these two cell populations. Moreover, the authors also found out that TCs are different from pericytes. A surprising finding of the study was that TCs are positive for mesenchymal marker CD29, which may imply the possibility that TCs give rise to cardiac mesenchymal cells [80].

## 6. Conclusions and Future Perspectives

TCs are a remarkable cell population whose potential is enormous from many different perspectives. They are possibly the “last piece of the puzzle” in understanding the etiopathogenesis of many diseases, including the elucidation of the processes occurring during the development of cardiovascular diseases. TCs provide clues for a more detailed and elaborated grasp of how the processes during cardiovascular regeneration unfold. Transplantation of TCs, either solitary or in tandem with stem cells, might be an important therapeutic measure that will positively influence the unfavorable capacity of the heart to repair itself after disease or injury. However, there are still many unanswered questions and underresearched areas that have to be addressed in future experiments in order to routinely use TCs in cell therapy of cardiovascular diseases in human subjects. These include the following:

- The standardization of protocols for TCs isolation, cultivation, and transplantation (although standardized protocols for the isolation of cardiac TCs are yet to be established, Romano et al. previously successfully isolated TCs from human skin using a novel two-step immunomagnetic microbead-based cell separation, taking advantage of the CD34^+^/CD31^−^ immunophenotype of TCs [81]; this methodology could also be applied for selective purification of cardiac TCs in future research);
- Figuring out the applicability and extrapolation of the results of animal model studies to human medicine;
- Viability assessment of transplanted TCs;
- Quantitative optimization of transplanted cells for each patient;
- Cost-effectiveness analysis.

## References

[1] Jensen R.V., Hjortbak M.V., Bøtker H.E. (2020). Ischemic Heart Disease: An Update. Semin. Nucl. Med..

[2] Giacca M. (2020). Cardiac Regeneration After Myocardial Infarction: An Approachable Goal. Curr. Cardiol. Rep..

[3] Cui B., Zheng Y., Sun L., Shi T., Shi Z., Wang L., Huang G., Sun N. (2018). Heart Regeneration in Adult Mammals after Myocardial Damage. Acta Cardiol. Sin..

[4] Wen H., Peng L., Chen Y. (2021). The effect of immune cell-derived exosomes in the cardiac tissue repair after myocardial infarction: Molecular mechanisms and pre-clinical evidence. J. Cell. Mol. Med..

[5] Veress B., Ohlsson B. (2020). Spatial relationship between telocytes, interstitial cells of Cajal and the enteric nervous system in the human ileum and colon. J. Cell. Mol. Med..

[6] Popescu L.M., Faussone-Pellegrini M.S. (2010). TELOCYTES—A case of serendipity: The winding way from Interstitial Cells of Cajal (ICC), via Interstitial Cajal-Like Cells (ICLC) to TELOCYTES. J. Cell. Mol. Med..

[7] Popescu L.M., Hinescu M.E., Ionescu N., Ciontea S.M., Cretoiu D., Ardelean C. (2005). Interstitial cells of Cajal in pancreas. J. Cell. Mol. Med..

[8] Popescu L.M., Hinescu M.E., Radu E., Ciontea S.M., Cretoiu D., Leabu M., Ardeleanu C. (2005). CD117/c-kit positive interstitial (Cajal-like) cells in human pancreas. J. Cell. Mol. Med..

[9] Cretoiu S.M., Popescu L.M. (2014). Telocytes revisited. Biomol. Concepts.

[10] Kondo A., Kaestner K.H. (2019). Emerging diverse roles of telocytes. Development.

[11] Zhang H., Yu P., Zhong S., Ge T., Peng S., Guo X., Zhou Z. (2016). Telocytes in pancreas of the Chinese giant salamander (Andrias davidianus). J. Cell. Mol. Med..

[12] Nicolescu M.I., Popescu L.M. (2012). Telocytes in the interstitium of human exocrine pancreas: Ultrastructural evidence. Pancreas.

[13] Xiao J., Wang F., Liu Z., Yang C. (2013). Telocytes in liver: Electron microscopic and immunofluorescent evidence. J. Cell. Mol. Med..

[14] Cretoiu S.M. (2016). Immunohistochemistry of Telocytes in the Uterus and Fallopian Tubes. Adv. Exp. Med. Biol..

[15] Liu T., Wang S., Li Q., Huang Y., Chen C., Zheng J. (2016). Telocytes as potential targets in a cyclophosphamide-induced animal model of premature ovarian failure. Mol. Med. Rep..

[16] Abd-Elhafeez H.H., Soliman S.A. (2017). New Description of Telocyte Sheaths in the Bovine Uterine Tube: An Immunohistochemical and Scanning Microscopic Study. Cells Tissues Organs.

[17] Ceafalan L., Gherghiceanu M., Popescu L.M., Simionescu O. (2012). Telocytes in human skin--are they involved in skin regeneration?. J. Cell. Mol. Med..

[18] Qi G., Lin M., Xu M., Manole C.G., Wang X., Zhu T. (2012). Telocytes in the human kidney cortex. J. Cell. Mol. Med..

[19] Luesma M.J., Gherghiceanu M., Popescu L.M. (2013). Telocytes and stem cells in limbus and uvea of mouse eye. J. Cell. Mol. Med..

[20] Hussein M.M., Mokhtar D.M. (2018). The roles of telocytes in lung development and angiogenesis: An immunohistochemical, ultrastructural, scanning electron microscopy and morphometrical study. Dev. Biol..

[21] Pasternak A., Gil K., Matyja A. (2016). Telocytes: New Players in Gallstone Disease. Adv. Exp. Med. Biol..

[22] Vannucchi M.G., Traini C., Manetti M., Ibba-Manneschi L., Faussone-Pellegrini M.S. (2013). Telocytes express PDGFRα in the human gastrointestinal tract. J. Cell. Mol. Med..

[23] Vannucchi M.G., Traini C. (2016). Interstitial cells of Cajal and telocytes in the gut: Twins, related or simply neighbor cells?. Biomol. Concepts.

[24] Vannucchi M.G. (2020). The Telocytes: Ten Years after Their Introduction in the Scientific Literature. An Update on Their Morphology, Distribution, and Potential Roles in the Gut. Int. J. Mol. Sci..

[25] Vannucchi M.G., Traini C., Guasti D., Giulio D.P., Faussone-Pellegrini M.S. (2014). Telocytes subtypes in human urinary bladder. J. Cell. Mol. Med..

[26] Wagener M.G., Leonhard-Marek S., Häger J.D., Pfarrer C. (2018). CD117- and vimentin-positive telocytes in the bovine teat sphincter. Anat. Histol. Embryol..

[27] Popescu L.M., Curici A., Wang E., Zhang H., Hu S., Gherghiceanu M. (2015). Telocytes and putative stem cells in ageing human heart. J. Cell. Mol. Med..

[28] Shim W. (2016). Myocardial Telocytes: A New Player in Electric Circuitry of the Heart. Adv. Exp. Med. Biol..

[29] Tay H., Vandecasteele T., Van den Broeck W. (2017). Identification of telocytes in the porcine heart. Anat. Histol. Embryol..

[30] Popescu L.M., Gherghiceanu M., Suciu L.C., Manole C.G., Hinescu M.E. (2011). Telocytes and putative stem cells in the lungs: Electron microscopy, electron tomography and laser scanning microscopy. Cell Tissue Res..

[31] Cretoiu D., Hummel E., Zimmermann H., Gherghiceanu M., Popescu L.M. (2014). Human cardiac telocytes: 3D imaging by FIB-SEM tomography. J. Cell. Mol. Med..

[32] Cretoiu S.M., Cretoiu D., Marin A., Radu B.M., Popescu L.M. (2013). Telocytes: Ultrastructural, immunohistochemical and electrophysiological characteristics in human myometrium. Reproduction.

[33] Díaz-Flores L., Gutiérrez R., García M.P., Sáez F.J., Díaz-Flores L., Valladares F., Madrid J.F. (2014). CD34^+^ stromal cells/fibroblasts/fibrocytes/telocytes as a tissue reserve and a principal source of mesenchymal cells. Location, morphology, function and role in pathology. Histol. Histopathol..

[34] Zhou Q., Wei L., Zhong C., Fu S., Bei Y., Huică R.I., Wang F., Xiao J. (2015). Cardiac telocytes are double positive for CD34/PDGFR-α. J. Cell. Mol. Med..

[35] Lis G.J., Dubrowski A., Lis M., Solewski B., Witkowska K., Aleksandrovych V., Jasek-Gajda E., Hołda M.K., Gil K., Litwin J.A. (2020). Identification of CD34^+^/PGDFRα^+^ Valve Interstitial Cells (VICs) in Human Aortic Valves: Association of Their Abundance, Morphology and Spatial Organization with Early Calcific Remodeling. Int. J. Mol. Sci..

[36] Gherghiceanu M., Manole C.G., Popescu L.M. (2010). Telocytes in endocardium: Electron microscope evidence. J. Cell. Mol. Med..

[37] Gherghiceanu M., Popescu L.M. (2010). Cardiomyocyte precursors and telocytes in epicardial stem cell niche: Electron microscope images. J. Cell. Mol. Med..

[38] Popescu L.M., Gherghiceanu M., Manole C.G., Faussone-Pellegrini M.S. (2009). Cardiac renewing: Interstitial Cajal-like cells nurse cardiomyocyte progenitors in epicardial stem cell niches. J. Cell. Mol. Med..

[39] Popescu L.M., Manole C.G., Gherghiceanu M., Ardelean A., Nicolescu M.I., Hinescu M.E., Kostin S. (2010). Telocytes in human epicardium. J. Cell. Mol. Med..

[40] Rosa I., Marini M., Manetti M. (2021). Telocytes: An Emerging Component of Stem Cell Niche Microenvironment. J. Histochem. Cytochem..

[41] Nagy E., Jermendy A.L., Merkely B., Maurovich-Horvat P. (2017). Clinical importance of epicardial adipose tissue. Arch. Med. Sci..

[42] Nerlekar N., Thakur U., Lin A., Koh J.Q.S., Potter E., Liu D., Muthalaly R.G., Rashid H.N., Cameron J.D., Dey D. (2020). The Natural history of Epicardial Adipose Tissue Volume and Attenuation: A long-term prospective cohort follow-up study. Sci. Rep..

[43] Christensen R.H., von Scholten B.J., Lehrskov L.L., Rossing P., Jørgensen P.G. (2020). Epicardial adipose tissue: An emerging biomarker of cardiovascular complications in type 2 diabetes?. Ther. Adv. Endocrinol. Metab..

[44] Díaz-Flores L., Gutiérrez R., García M.P., González-Gómez M., Carrasco J.L., Alvarez-Argüelles H., Díaz-Flores L. (2020). Telocytes/CD34+ Stromal Cells in Pathologically Affected White Adipose Tissue. Int. J. Mol. Sci..

[45] Zhao B., Chen S., Liu J., Yuan Z., Qi X., Qin J., Zheng X., Shen X., Yu Y., Qnin T.J. (2013). Cardiac telocytes were decreased during myocardial infarction and their therapeutic effects for ischaemic heart in rat. J. Cell. Mol. Med..

[46] Liskova Y.V., Stadnikov A.A., Salikova S.P. (2018). The role of telocytes in myocardial remodeling and the development of cardiovascular complications in patients with chronic heart failure after coronary artery bypass grafting. Kardiologiia.

[47] Lin Y.K., Chen Y.J. (2019). Telocytes: Supporting cells participating in ventricular arrhythmogenesis?. J. Arrhythm..

[48] Mandache E., Gherghiceanu M., Macarie C., Kostin S., Popescu L.M. (2010). Telocytes in human isolated atrial amyloidosis:ultrastructural remodelling. J. Cell. Mol. Med..

[49] Nour M.S., Sarhan N.R., Mazroa S.A., Gawish S.A. (2017). Histological and immunohistochemical study of cardiac telocytes in a rat model of isoproterenol-induced myocardial infarction with a reference to the effect of grape seed extract. Acta Histochem..

[50] Varga I., Kyselovic J., Danihel L., Klein M., Barczi T., Galfiova P., Danisovic L. (2017). Cardiac telocytes as principal interstitial cells for myocardial reparation and regeneration after infarction—Our hope. Bratisl. Lek. Listy.

[51] Jenča D., Melenovský V., Stehlik J., Staněk V., Kettner J., Kautzner J., Adámková V., Wohlfahrt P. (2021). Heart failure after myocardial infarction: Incidence and predictors. ESC Heart Failure.

[52] Richter M., Kostin S. (2015). The failing human heart is characterized by decreased numbers of telocytes as result of apoptosis and altered extracellular matrix composition. J. Cell. Mol. Med..

[53] Manetti M., Rosa I., Messerini L., Ibba-Manneschi L. (2015). Telocytes are reduced during fibrotic remodelling of the colonic wall in ulcerative colitis. J. Cell. Mol. Med..

[54] Czubryt M.P. (2019). Cardiac Fibroblast to Myofibroblast Phenotype Conversion-An Unexploited Therapeutic Target. J. Cardiovasc. Dev. Dis..

[55] Manetti M., Rosa I., Messerini L., Guiducci S., Matucci-Cerinic M., Ibba-Manneschi L. (2014). A loss of telocytes accompanies fibrosis of multiple organs in systemic sclerosis. J. Cell. Mol. Med..

[56] Banciu D.D., Banciu A., Radu B.M. (2016). Electrophysiological Features of Telocytes. Adv. Exp. Med. Biol..

[57] DeSimone C.V., McLeod C.J., Gomez Pinilla P.J., Beyder A., Farrugia G., Asirvatham S.J., Kapa S. (2019). Telocytes express ANO-1-encoded chloride channels in canine ventricular myocardium. J. Arrhythm..

[58] Mitrofanova L.B., Gorshkov A.N., Konovalov P.V., Krylova J.S. (2018). Telocytes in the human sinoatrial node. J. Cell. Mol. Med..

[59] Gherghiceanu M., Hinescu M.E., Andrei F., Mandache E., Macarie C.E., Faussone-Pellegrini M.S., Popescu L.M. (2008). Interstitial Cajal-like cells (ICLC) in myocardial sleeves of human pulmonary veins. J. Cell. Mol. Med..

[60] Bei Y., Zhou Q., Sun Q., Xiao J. (2016). Telocytes in cardiac regeneration and repair. Semin. Cell Dev. Biol..

[61] Faussone-Pellegrini M.S., Bani D. (2010). Relationships between telocytes and cardiomyocytes during pre- and post-natal life. J. Cell. Mol. Med..

[62] Sukhacheva T.V., Nizyaeva N.V., Samsonova M.V., Chernyaev A.L., Shchegolev A.I., Serov R.A. (2020). Telocytes in the Myocardium of Children with Congenital Heart Disease Tetralogy of Fallot. Bull. Exp. Biol. Med..

[63] Liao Z., Chen Y., Duan C., Zhu K., Huang R., Zhao H., Hintze M., Pu Q., Yuan Z., Lv L. (2021). Cardiac telocytes inhibit cardiac microvascular endothelial cell apoptosis through exosomal miRNA-21-5p-targeted cdip1 silencing to improve angiogenesis following myocardial infarction. Theranostics.

[64] Cismaşiu V.B., Popescu L.M. (2015). Telocytes transfer extracellular vesicles loaded with microRNAs to stem cells. J. Cell. Mol. Med..

[65] Marini M., Ibba-Manneschi L., Manetti M. (2017). Cardiac Telocyte-Derived Exosomes and Their Possible Implications in Cardiovascular Pathophysiology. Adv. Exp. Med. Biol..

[66] Popescu L.M., Fertig E.T., Gherghiceanu M. (2016). Reaching out: Junctions between cardiac telocytes and cardiac stem cells in culture. J. Cell. Mol. Med..

[67] Lv L., Liao Z., Luo J., Chen H., Guo H., Yang J., Huang R., Pu Q., Zhao H., Yuan Z. (2020). Cardiac telocytes exist in the adult Xenopus tropicalis heart. J. Cell. Mol. Med..

[68] Zheng L., Li L., Qi G., Hu M., Hu C., Wang S., Li J., Zhang M., Zhang W., Zeng Y. (2018). Transplantation of Telocytes Attenuates Unilateral Ureter Obstruction-Induced Renal Fibrosis in Rats. Cell. Physiol. Biochem..

[69] Zhang D., Song D., Shi L., Sun X., Zheng Y., Zeng Y., Wang X. (2020). Mechanisms of interactions between lung-origin telocytes and mesenchymal stem cells to treat experimental acute lung injury. Clin. Transl. Med..

[70] Ibba-Manneschi L., Rosa I., Manetti M. (2016). Telocytes in Chronic Inflammatory and Fibrotic Diseases. Adv. Exp. Med. Biol..

[71] Miao Q., Shim W., Tee N., Lim S.Y., Chung Y.Y., Ja K.P., Ooi T.H., Tan G., Kong G., Wei H. (2014). iPSC-derived human mesenchymal stem cells improve myocardial strain of infarcted myocardium. J. Cell. Mol. Med..

[72] Ja K.P., Miao Q., Zhen Tee N.G., Lim S.Y., Nandihalli M., Ramachandra C.J.A., Mehta A., Shim W. (2016). iPSC-derived human cardiac progenitor cells improve ventricular remodelling via angiogenesis and interstitial networking of infarcted myocardium. J. Cell. Mol. Med..

[73] FIPAT (2008). Terminologia Histologica: International Terms for Human Cytology and Histology.

[74] Varga I., Gálfiová P., Blanková A., Konarik M., Báča V., Dvořákova V., Musil V., Turyna R., Klein M. (2019). Terminologia Histologica 10 years on: Some disputable terms in need of discussion and recent developments. Ann. Anat..

[75] Rusu M.C., Hostiuc S. (2019). Critical review: Cardiac telocytes vs cardiac lymphatic endothelial cells. Ann. Anat..

[76] Rosa I., Marini M., Sgambati E., Ibba-Manneschi L., Manetti M. (2020). Telocytes and lymphatic endothelial cells: Two immunophenotypically distinct and spatially close cell entities. Acta Histochem..

[77] Rosa I., Taverna C., Novelli L., Marini M., Ibba-Manneschi L., Manetti M. (2019). Telocytes constitute a widespread interstitial meshwork in the lamina propria and underlying striated muscle of human tongue. Sci. Rep..

[78] Hostiuc S., Marinescu M., Costescu M., Aluaș M., Negoi I. (2018). Cardiac telocytes. From basic science to cardiac diseases. II. Acute myocardial infarction. Ann. Anat..

[79] Varga I., Kyselovič J., Danišovič Ľ., Gálfiová P., Kachlík D., Polák Š., Klein M. (2019). Recently discovered interstitial cells termed telocytes: Distinguishing cell-biological and histological facts from fictions. Biologia.

[80] Bei Y., Zhou Q., Fu S., Lv D., Chen P., Chen Y., Wang F., Xiao J. (2015). Cardiac telocytes and fibroblasts in primary culture: Different morphologies and immunophenotypes. PLoS ONE.

[81] Romano E., Rosa I., Fioretto B.S., Lucattelli E., Innocenti M., Ibba-Manneschi L., Matucci-Cerinic M., Manetti M. (2020). A Two-Step Immunomagnetic Microbead-Based Method for the Isolation of Human Primary Skin Telocytes/CD34+ Stromal Cells. Int. J. Mol. Sci..

